# Comparison of the Direct Oral Anticoagulants and Warfarin in Patients With Atrial Fibrillation and Valvular Heart Disease: Updated Systematic Review and Meta-Analysis of Randomized Controlled Trials

**DOI:** 10.3389/fcvm.2021.712585

**Published:** 2021-09-22

**Authors:** Yasmin de Souza Lima Bitar, Andre Rodrigues Duraes, Leonardo Roever, Mansueto Gomes Neto, Liliane Lins-Kusterer, Edimar Alcides Bocchi

**Affiliations:** ^1^Post-graduate Program in Medicine and Health (PPgMS)/Federal University of Bahia (UFBA), Salvador, Brazil; ^2^Federal University of Bahia, UFBA, Salvador, Brazil; ^3^Federal University of Uberlandia, Uberlândia, Brazil; ^4^University of São Paulo Medical School, HCFMUSP, São Paulo, Brazil; ^5^Heart Institute, InCor, São Paulo, Brazil

**Keywords:** valvular atrial fibrillation, valvular heart disease, warfarin, direct oral anticoagulants, anticoagulation

## Abstract

**Background:** Direct oral anticoagulants (DOACS) are approved for use in non-valvular atrial fibrillation (AF). This systematic review and meta-analysis aimed to evaluate the efficacy and safety of DOACs vs. warfarin and update the evidence for treatment of AF and valvular heart disease (VHD).

**Methods:** We identified randomized clinical trials (RCTs) and *post-hoc* analyses comparing the use of DOACS and Warfarin in AF and VHD, including biological and mechanical heart valves (MHV), updating from 2010 to 2020. Through systematic review and meta-analysis, by using the “Rev Man” program 5.3, the primary effectiveness endpoints were stroke and systemic embolism (SE). The primary safety outcome was major bleeding, while the secondary outcome included intracranial hemorrhage. We performed prespecified subgroup analyses. Data were analyzed by risk ratio (RR) and 95% confidence interval (CI) and the I-square (*I*^2^) statistic as a quantitative measure of inconsistency. Risk of bias and methodological quality assessment of included trials was evaluated with the modified Cochrane risk-of-bias tool.

**Results:** We screened 326 articles and included 8 RCTs (*n* = 14.902). DOACs significantly reduced the risk of stroke/SE (RR 0.80, 95% CI: 0.68–0.94; *P* = 0.008; moderate quality evidence; *I*^2^ = 2%) and intracranial hemorrhage (RR 0.40, 95% CI: 0.24–0.66; *P* = 0.0004; *I*^2^ = 49%) with a similar risk of major bleeding (RR 0.83, 95% CI: 0.56–1.24; *P* = 0.36; *I*^2^ = 88%) compared to Warfarin.

**Conclusions:** In this update, DOACs remained with similar efficacy and safety compared to warfarin in thromboprophylaxis for AF and VHD.

## Introduction

Direct oral anticoagulants (DOACs) have been developed as a alternative to vitamin k antagonist (VKA) and emerged as the preferred treatment option for atrial fibrillation (AF) in the general population (class I, LOE: B), as well as in the prophylaxis or treatment of deep vein thrombosis and pulmonary embolism (1). Prior studies in non-valvular AF using DOACs demonstrated their non-inferiority compared to the use of warfarin, but in some trials, superior (2).

Recent guidelines supported the use of DOACs in native valve diseases (CHA2DS2-VASc score of 2 or greater). However, in moderate to severe mitral stenosis (MS) and mechanical heart valves (MHV), the use of VKA in the prevention of thromboembolic events is the only established option (class I, LOE: A) (3), A low time in therapeutic range (TTR) was observed in MHV populations in use of warfarin, which can increase the cardiac and thromboembolic risk. (4) In contrast, the TTR control was even lower in use of acenocoumarol than warfarin in the same population. (5) In the presence of bioprosthetic valves (≥ 3 months postoperatively) and AF, non-vitamin K antagonist oral anticoagulants are a reasonable option to VKAs (class I, LOE: A) (6).

Previous meta-analyses compared DOACs to warfarin for the treatment of AF and valvular heart disease (VHD). The study aggregated data from 4 or 5–of the current eight available–randomized controlled trials (RCTs), suggesting that DOACs have similar safety and efficacy compared to VKA (7–9). Based on this knowledge gap, this systematic review and updated meta-analysis aimed to compare the efficacy and safety of DOACs vs. warfarin in adult patients with AF and VHD by aggregating results from all available RCTs and to assess their relative benefit in specific subgroups.

## Methods

We conducted a systematic review of the literature and meta-analysis carried out to the standards established by the PRISMA recommendation (“Preferred Reporting Items for Systematic Reviews and Meta-Analyses”) (10). More details are available in Supplementary File 1 (Supplementary Table E1).

### Literature Research and Study Selection

The databases included PubMed, LILACS, MEDLINE, SciELO and Cochrane Library (November 2020—December 2020), with year restriction for 2010 to 2020. Researchers used predefined search terms combined with filters to identify RCTs (complete search strategy in Supplementary File 2. To be eligible for inclusion, studies had to fulfill all predefined inclusion criteria that were defined as follows: RCTs that compared DOACS (Dabigatran, Rivaroxaban, Apixaban Edoxaban, and/or Betrixaban) to Warfarin in adult humans (aged ≥18 years) with AF and VHD (including patients with bioprosthesis and MHV ≥ 3 months postoperatively). Exclusion criteria were as follows: articles not focused on the use of DOACS in VHD and AF; inclusion of patients <18 years of age; observational studies; non-randomized clinical trials; studies performed in animals; reviews; duplicate publications reporting the same trials.

### Data Collection

Two reviewers independently (B, Y, and D, A) evaluated the list of titles and abstracts from each data source. For articles considered eligible, researchers accessed the full text to verify if they met inclusion criteria, prior to data extraction. Any disagreements were resolved through a consensus discussion among reviewers. (More details in Supplementary File 2).

### Evaluated Outcomes

We considered the primary endpoint of efficacy, stroke composition and SE, while the primary safety outcome was the presence of major bleeding (according to the International Society of Thrombosis and Haemostasis—ISTH–definition) (11). Intracranial hemorrhage was characterized as a secondary outcome. Risk of bias and methodological quality assessment of included trials was evaluated with the modified Cochrane risk-of-bias tool (Supplementary File 2)–version 2 of the Cochrane risk-of-bias tool for randomized trials (RoB 2) (12). The assessment involved five items: risk of bias, imprecision, inconsistency, indirectness, and publication bias. The quality of evidence was downgraded by one level for risk of bias when more than a quarter of the studies included in meta-analysis were considered at high risk of bias (Studies without allocation concealment, random allocation, and/or sample size calculation). Results were considered imprecise if the pooled sample size was <300 for dichotomous or <400 for continuous outcomes, and inconsistent if the heterogeneity between RCTs was substantial (i.e., I^2^ > 40%).

### Statistical Analysis

Statistical analysis was performed using the Cochrane Collaboration Review Manager Software (RevMan version 5.3, 2011). We used the random-effects meta-analysis model, and forest plots were used to present the pooled stimates of the risk ratio (RR) and 95% confidence interval (CI). A *p*-value ≤ 0.05 was considered statistically significant. Researchers independently grouped, in different estimates, the studies that presented the use of different dosages, using the random-effects model in the meta-analysis. The I-square (*I*^2^) statistic was used as a quantitative measure of inconsistency. We used the GRADEpro software (Grading of Recommendations, Assessment, Development and Evaluation profiler) to assess the quality of evidence across studies and minimize bias in our findings and recommendations. GRADEpro classified the level of evidence as very low, low, moderate, or high (13).

## Results

**Figure 1:**
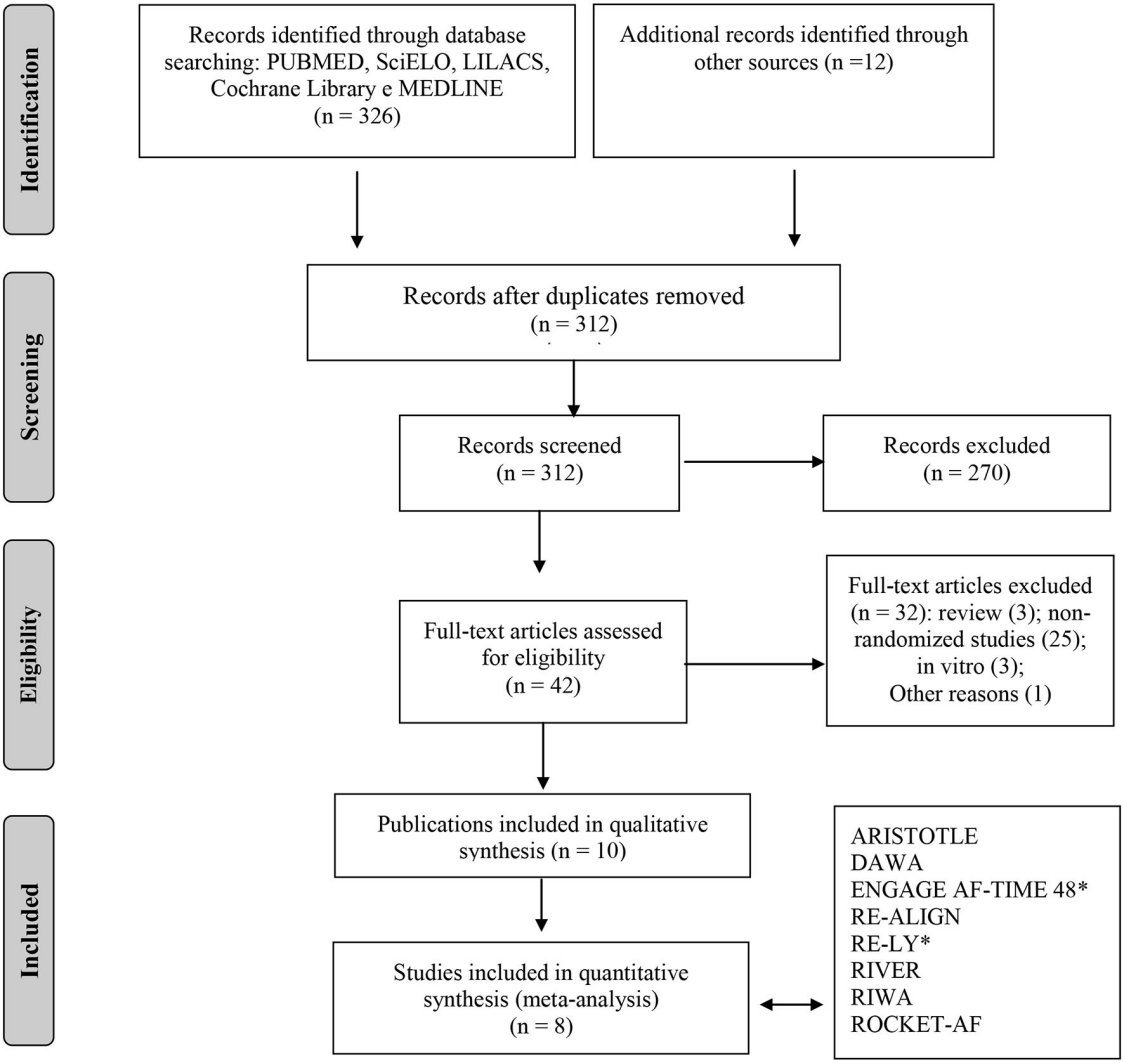
Flowchart of study selection adapted from the PRISMA recommendation (“Preferred Reporting Items for Systematic Reviews and Meta-Analyzes”). *For quantitative analysis, the study was separated into two subgroups (two different doses of NOACS was tested).

A total of 326 published records matched the predefined search terms. In our review, we identified a total of 10 studies that met eligibility criteria based on the screening process. Of those, eight were RCTs (*n* = 14.902), contributing to 10 different publications, including six publications on specific subgroups or *post hoc* analysis (14–21). In two studies, we found two different tested doses. Therefore, we performed specific analyses for each one. Figure 1 showed the Preferred Reporting Items for Systematic Reviews and Meta-Analyses (PRISMA) flow diagram, which summarizes the study selection process. Table 1 presented the different types of study design, outcomes, and VHD included in this analysis.

**Table 1:** Types of study design, outcomes and VHD included in the different trials.

**Trial**	**Design**	**Data**	**Arms**	**Outcomes** **DOAC vs. AVK**	**MHV**	**NVD**	**BP**	**VR**
Eikelboom et al. (14) (RE-ALIGN)	*Open-label RCT N = 252*	RCT data	Dabigatran 150, 220 or 300 mg BID (according to CrCl) or AVK dose-adjusted to INR.	Population B (≥3 months postoperative)–Stroke/SE, major and IH bleeding: none, both groups. Death, stroke/SE, AIT or myocardial infarction: 3 vs. 0 (HR 1.94 (0.64–5.86)	Yes	-	-	-
Breithardt et al. (15) (ROCKET-AF)	*DB RCT N = 1,945*	*Post hoc* retrospective RCT subgroups	Rivaroxaban 20 mg QD (or adjusted for 15 mg^*^) or AVK dose-adjusted to INR	Stroke/SE: 38 vs. 50 (HR 0.83, 95% CI 0.55–1.27); major bleeding: 88 vs. 68 (HR 1.56, 95% CI 1.14–2.14); IH bleeding: 13 vs. 12 (HR 1.27, 95% CI 0.58–2.79)	-	Yes	-	-
Avezum et al. (16) (ARISTOTLE)	DB RCT *N* = 4,008	*Post hoc* retrospective RCT subgroups	Apixaban 5 mg BID (or adjusted for 2.5 mg^**^) or AVK dose-adjusted to INR	Stroke/SE: 64 vs. 89 (HR 0.70, 95% CI 0.51–0.97); major bleeding: 99 vs. 119 (HR 0.79, 95% CI 0.61–1.04) and IH bleeding: 10 vs. 34 (HR 0.28, 95% CI 0.14–0.57)	-	Yes	Yes	Yes
Duraes et al. (21) (DAWA)	Open-label RCT *N* = 27	RCT data	Dabigatran 110 mg BID or AVK dose-adjusted to INR	Stroke/SE: 0 vs. 2 (RR 1.1, 95% CI 0.9–1.3); major bleeding: 1 vs. 2 (RR 2.8, 95% CI 0.2–35)	-	-	Yes	-
Ezekowitz et al. (18) (RE-LY)	Open-label RCT *N* = 3,950	*Post hoc* retrospective RCT subgroups	Dabigatran 110 mg BID or Dabigatran 150 mg BID or AVK dose-adjusted to INR	Stroke/SE: 47 (110 mg) vs. 30 (150 mg) vs. 49 (HR 0.97, 95% CI 0.65–1.45 and HR 0.59, 95% CI 0.37–0.97); major bleeding: 96 (110 mg) vs. 113 (150 mg) vs. 132 (HR 0.73, 95% CI 0.56–0.95 and (HR 0.82, 95% CI 0.64–1.06; IH bleeding: 7 (110 mg) vs. 9 (150) vs. 24 (HR 0.29, 95% CI 0.13–0.68 and HR 0.36, 95% CI 0.17–0.77)	-	Yes	-	-
De Caterina et al. (19) (ENGAGE AF-TIME 48)	DB RCT *N* = 2,824	*Post hoc* retrospective RCT subgroups	Edoxaban 60 mg QD (or 30 mg^***^) or Edoxaban 30 mg QD (or 15 mg^***^) or AVK dose-adjusted to INR	Stroke/SE: 49 (30 mg) vs. 33 (60 mg) vs. 50 (HR 0.97, 95% CI 0.66–1.44 and HR 0.69, 95% CI 0.44–1.07); major bleeding: 38 (30 mg) e 61 (60 mg) vs. 89 (HR 0.41, 95% CI 0.28–0.60 and HR 0.74, 95% CI 0.53–1.02); IH bleeding: 5 (30 mg) vs. 6 (60 mg) vs. 7 (HR 0.29, 95% CI 0.11–0.79 and HR 0.63, 95% CI 0.23–1.73	-	Yes	Yes	Yes
Durães et al. (17) (RIWA)	Open-label RCT *N* = 44	RCT data	Rivaroxaban 15 mg BID or Warfarin according to INR	Stroke/SE: 1 vs. 3 (HR 0.27, 95% CI 0.02–2.85); minor bleeding 6 vs. 6 (HR 0.88, 95% CI 0.23–3.32).	Yes	-	-	-
Guimarães et al. (20) (RIVER)	*Open-label RCT N = 1,005*	RCT data	Rivaroxaban 20 mg QD (or 15 mg if CrCl of 30–49 ml/min) or AVK dose-adjusted to INR	Stroke/SE: 3 vs. 12 (HR 0.25, 95% CI 0.07–0.88); major bleeding: 7 vs. 13 (HR 0.54, 95% CI 0.21–1.35); IH bleeding: 0 vs. 5.	-	-	Yes	-

*BID, twice daily; QD, once a day; DB, double-blinded; INR, International Normalized Ratio; IH, intracranial hemorrhage; MHV, mechanical heart valve; RCT, randomized controlled trial; VKA, INR-adjusted vitamin K antagonist; AF, atrial fibrillation; DOAC, direct oral anticoagulants; CrCl, creatinine clearence; P-gp, P-glycoprotein; NVD, native valve disease.*

* *If CrCl 30–49 mL/min;*

** *≥2 of: age ≥80 years, weight ≤ 60 kg, Cr ≥ 1.5 mg/dL);*

*** *QD if CrCl 30–50 mL/min, ≤ 60 kg, or concomitant P-gp inhibitors*.

### Study Characteristics

Among the included studies, we identified phase II (3) and III (5) RCTs (see more details in Supplementary Tables E1, 2). Three publications evaluated the use of Dabigatran (the RE-ALIGN study—with MHV only (14), *post-hoc* analysis of the RE-LY study (16) and the DAWA study (17)—with bioprosthetic valves only). The remaining studies are as follows: one evaluated the use of Apixaban by the *post-hoc* study of ARISTOTLE (16), three evaluated the use of Rivaroxaban (*post-hoc* analysis of the ROCKET-AF study (15), the RIWA study (21)—with MHV and the RIVER study (20)—with bioprosthetic valves in mitral position only) and one analyzed the use of Edoxaban (*post-hoc* analysis of the ENGAGE AF-TIME-48 trial) (19). We did not identify RCTs comparing betrixaban with warfarin and VHD.

The main clinical characteristics and risk factors for bleeding and (thromboembolic events) TE in patients with AF and VHD are detailed in Supplementary Table E2. The most frequent subtype of VHD identified in the enrolled population in these studies were as follows: 7.842 individuals with MR and aortic regurgitation (AR) 2.559, 3.303 with TR, 1.235 with AS, 708 with MS, 393 had some type of valve repair or repair, 296 patients with MHV and 1.223 with bioprostheses.

### Outcomes: Stroke/SES, Major Bleeding, and Intracranial Hemorrhage

**Figure 2:**
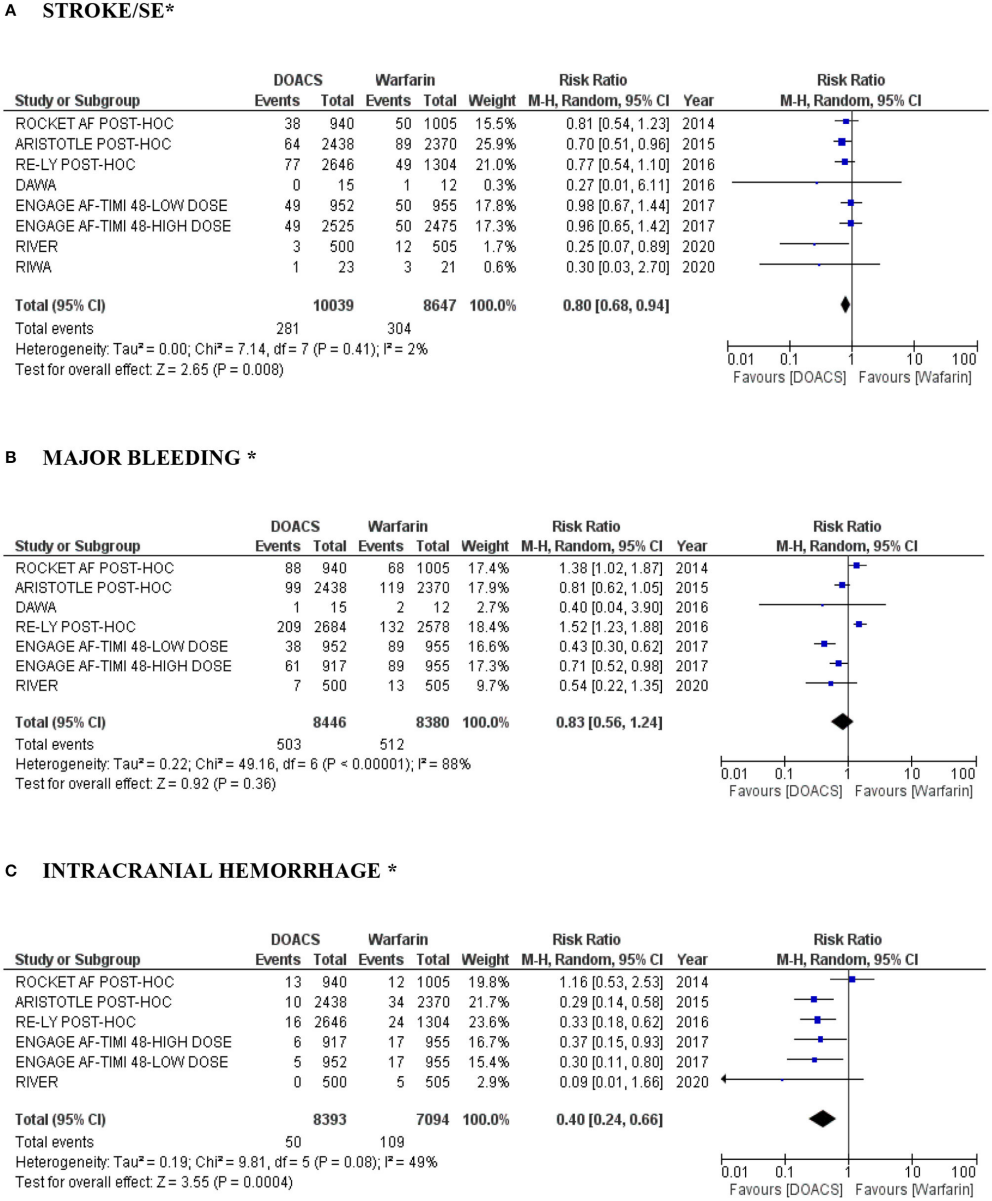
**(A–C)** “Forest plot” with individual and pooled estimates of the risk of stroke/SE, major bleeding and intracranial hemorrhage in patients with VHD and AF using DOACs (at different dosages) compared to warfarin. A random effects model was used to establish RR and 95% CI; SE, systemic embolism; AF, atrial fibrillation; VHD, valvular heart disease; DOAC, direct oral anticoagulant. *In the RE-ALIGN study performed by Eikelboom et al. (14), events in stroke/major bleeding/intracranial hemorrhage were not reported in the population B (MHV ≥ 3 months postoperatively). In addition, it was not possible to perform analysis by dose of the RE-LY study *post-hoc* performed by Ezekowitz et al. (18) (lack of data).

DOACs were associated with a lower risk of stroke and SE in patients with VHD and AF (RR 0.80, 95% CI: 0.68–0.94; *P* = 0.008; moderate quality evidence) (Figure 2A). Heterogeneity among the studies evaluated was low (*I*^2^ = 2). Major bleeding was numerically lower among the DOAC 002group, showed a favorable effect of its use compared with warfarin (RR 0.83, 95% CI: 0.56–1.24; *P* = 0.36; low quality evidence), with *I*^2^ calculated at 88% (*P* < 0.00001), demonstrating high heterogeneity (Figure 2B). On the other hand, DOACs were associated with a significant reduction in the risk of intracranial hemorrhage in patients with VHD and AF in comparison with warfarin (RR 0.40, 95% CI: 0.24–0.66; *P* = 0.0004; low quality evidence), with an estimated *I*^2^ of 49% (*P* = 0.08) demonstrating moderate heterogeneity (Figure 2C).

### Subgroup Analyses (Bioprosthetic Heart Valves)

**Figure 3:**
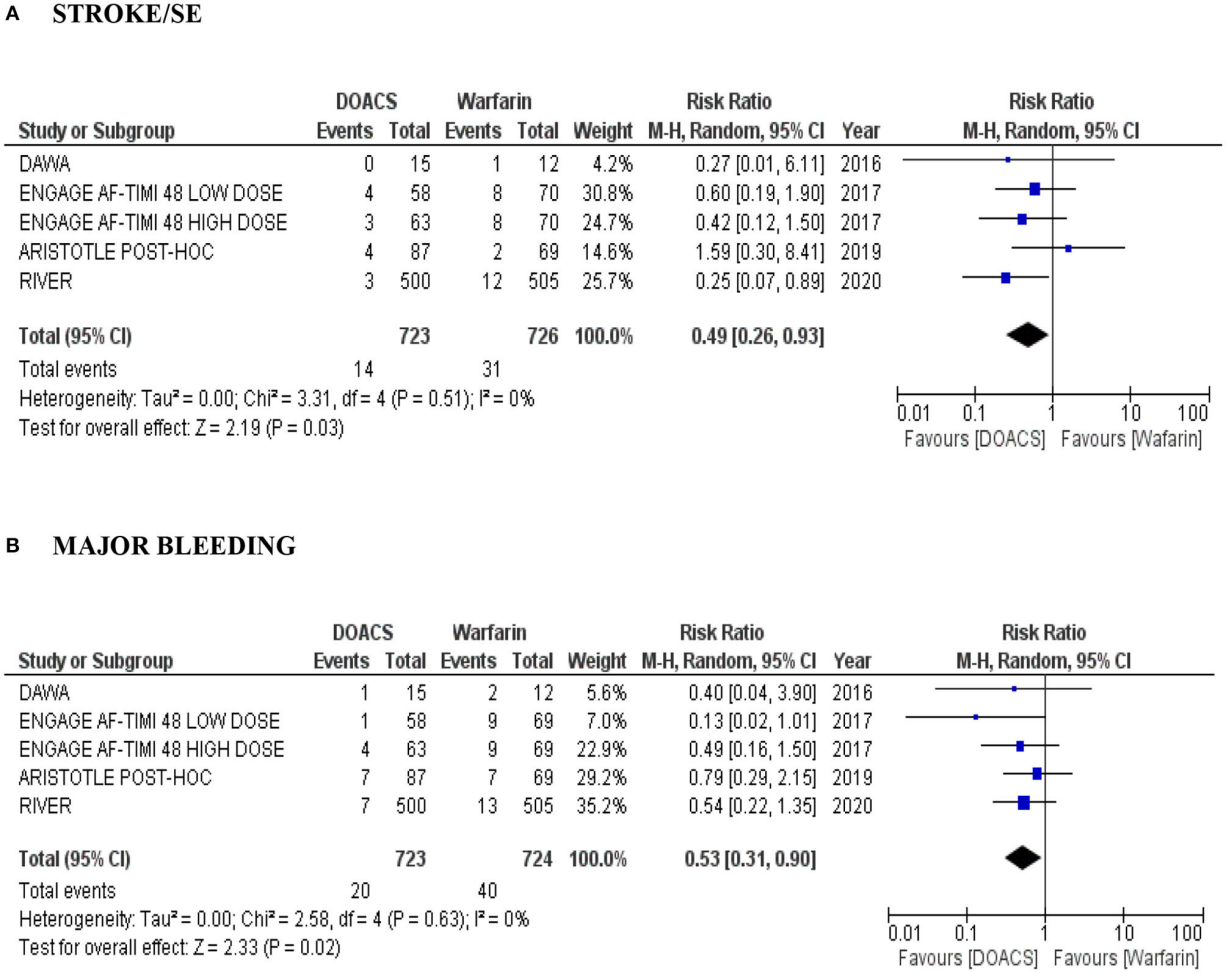
**(A,B)** Forest plot with pooled estimates of the risk of stroke/SE and major bleeding and intracranial hemorrhage in patients with AF and bioprosthesis with DOACs (at different dosages) compared to warfarin. CI, confidence interval; SE, systemic embolism; AF, atrial fibrillation; OR, odds ratio; VHD, valvular heart disease; DOAC, direct oral anticoagulant; A random effects model was used to establish RR and 95% CI.

We conducted a subgroup analysis of the risk of stroke, SE and major bleeding in patients with bioprosthetic valves and AF treated with DOACs compared to warfarin. There were four studies (overall 1.449 patients), with detailed data about this subgroup: another *post hoc* analysis of ARISTOTLE trial, this time, with only bioprosthesis (87 in the apixaban arm vs. 69 in the warfarin arm) (22) and of the ENGAGE AF-TIMI 48 study (high and low dose edoxaban vs. warfarin) (23), a pilot DAWA study (15 in the dabigatran arm vs. 12 in the warfarin arm) (17) and the RIVER study (500 in the rivaroxaban arm vs. 505 in the warfarin arm) (20). In our analyses, DOACs were more effective than warfarin with lower risk of stroke and SE (RR 0.49, 95% CI: 0.26–0.93; *P* = 0.03; moderate quality evidence) (Figure 3A) and with a lower risk of major bleeding (RR 0.53, 95% CI: 0.31–0.90; *P* = 0.02; moderate quality evidence) (Figure 3B). Heterogeneity among the studies evaluated was low (*I*^2^ = 0%).

### Risk of Bias Across Studies and Quality of Evidence

The overall risk of reporting bias was low based on our analysis using the Cochrane Collaboration Tool (details in Supplementary File 5 and Supplementary Figure E1). The quality of evidence according to the GRADE system is presented in Supplementary File 6 (Supplementary Table E3). We summarize the main pharmacological characteristics and indications of DOACs approved by the FDA for use in the United States (U.S.) to date, (see more details in Supplementary Table E4).

## Discussion

The main findings from the our pooled analyses were: (i) DOACs significantly reducing the risk of stroke/systemic embolism and intracranial hemorrhage, even after the inclusion of patients with MHV ≥ 3 months postoperatively; (ii) the overall risk of major bleeding was lower; and (iii) the difference for stroke/systemic embolism and major bleeding was persistent in a subgroup analysis of bioprosthetic valves and AF. Prior systematic reviews and meta-analyses in the same direction, suggesting that DOACs are effective as warfarin in reducing the risk of TE in AF-associated VHD with a lower association with major bleeding (7, 8, 24). The robustness of our results was higher for intracranial hemorrhage followed by Stroke/SE.

In the context of heart valve surgery, the choice for MHV emerges as an attractive possibility due to its higher durability in comparison with bioprosthetic valves. On the other hand, the need for long-term anticoagulation exclusively with the use of VKA is relatively complex. There is the need for dose adjustment by laboratory hemostatic parameters, the possibility of interactions with nutrients, and drugs, which difficults the management of these drugs in clinical practice. These factors contribute to the instability of the international normalized ratio (INR), increasing the risks of thromboembolic and hemorrhagic disorders (25).

Small studies and *post-hoc* analyses of large RCTs presented promising results regarding the superiority of DOACs use compared to warfarin in the presence of bioprosthesis and AF in non-valvar AF. Based on these results, the RIVER study (Rivaroxaban in Patients with Atrial Fibrillation and a Bioprosthetic Mitral Valve) was a large randomized trial, prospective, non-inferiority, open-label, involving the enrollment of 1,005 individuals with 1-year follow-up (20). In this study, the authors identified a higher incidence of thrombotic events and major bleeding in the warfarin group, despite the absence of associated statistical significance. Rivaroxaban has not been inferior in this scenario.

Since 2015, the European Heart Rhythm Association (EHRA) stated that patients with AF and bioprosthesis (>3 months postoperative) could be eligible for DOACs (26). However, this use is still controversial. Studies to date show promising data for DOACs use in patients with bioprosthesis and AF. Future analyses are needed to evaluate the safety and efficacy of their use in other forms of VHD (eg, MS moderate to severe), including in patients with MHV. The current recommendations of the AHA/ACC and ESC/EACTS (2017) maintain the use of VKA until further studies can elucidate the safety and efficacy of DOACs in this population (27, 28).

After encouraging results from previously published preclinical studies and considering the high prevalence rates of MS in Asian countries, associated with the presence of high rates of intracranial hemorrhage in individuals using VKA, the University of Hong Kong is conducting the DAVID-MS study (DAbigatran for Stroke PreVention In Atrial Fibrillation in MoDerate or Severe Mitral Stenosis The DAVID-MS study is a randomized, prospective, open, phase IV study, which aims to evaluate the efficacy and safety of the use of Dabigatran (150 mg or 110 mg according to creatinine clearance level, twice daily) compared with warfarin (targeting to INR 2–3) for preventing thromboembolism in individuals diagnosed with AF associated with moderate to severe MS (29).

Based on the finds of preclinical studies, and the RE-ALIN (Dabigatran vs. Warfarin in Patients with Mechanical Heart Valves) study (14), the ideal candidate for the use of DOACs (especially FXa inhibitors) in MHV (recognized for its fundamental action activating the intrinsic coagulation pathway by contact) could be considered in case of postoperative valve replacement (>3 months), aortic position (minor thrombogenicity), absence of systolic dysfunction, low risk of bleeding, absence of hypercoagulability states, and good drug adherence (25).

An experimental model with MHV, using an *in vitro* thrombosis tester, evaluated the efficacy of dabigatran as compared to unfractionated heparin (UFH) and low-molecular-weight heparin (LMWH) in thromboembolism prevention (30). Dabigatran was similarly effective in preventing clot formation compared to UFH and LMWH. However, serum levels 10 times higher than the RE-ALIGN trial doses. Therefore, the clinical application is not possible due to the risk of serious adverse effects concerning the use of warfarin. Other studies with MHV, using *in vitro* or animal models, demonstrated the efficacy of DOACs. Both dabigatran and rivaroxaban prevented thrombus formation as enoxaparin (31–33).

In this scenario, where is a large knowledge gap, new studies involving humans' subjects are required to evaluate the applicability of the use of Factor Xa (FXa) inhibitors in MHV, for the feasibility of developing a new phase III RCTs. The PROACT Xa study is being conducted aiming to determine if patients with a mechanical On-X aortic heart valve or On-X ascending aortic prosthesis can be maintained safely on apixaban (5 mg twice daily) in comparison to warfarin (INR range of 2.0–3.0). The PROACT Xa is a multicenter, prospective, and open-label study, which is in the phase of recruitment. The study estimates the randomized enrollment of 1,000 patients from 60 sites in North America who underwent aortic valve replacement at least 3 months prior, with an expected duration of 2 years of follow-up (34). The RENOVATE study (Randomized, Evaluation of Long-term Anticoagulation With Oral Factor Xa Inhibitor vs. Vitamin K Antagonist After Mechanical Aortic Valve Replacement) is also being conducted. It is a prospective Korean study (Asan Medical Center), open-label, phase IV, estimating the enrollment of 1,374 participants, which is not recruiting participants yet (35).

Lastly, FDA approved (2018) the use of betrixaban (a new FXa Inhibitor) for extended thromboprophylaxis, based on the results of the APEX study (Extended Thromboprophylaxis with Betrixaban in Acutely Ill Medical Patients), a randomized, phase III, double-blind, double-dummy, active-controlled, and multinational clinical trial. In this study, the authors identified that extended prophylaxis with betrixaban led to a reduction in VTE compared with standard-duration enoxaparin, without an increase of hemorrhagic events (36). Betrixaban has the longest half-life among the DOACs, with an effective half-life of 19–27 h, and is mainly cleared via the hepatobiliary system. Therefore, its use is possible in case of severe renal insufficiency (37). To date, there is still no report of its use in valvular AF.

### Limitations of the Study

Our updated meta-analysis has several limitations. First, most of our results were produced through information obtained in *post-hoc* analyses of large RCTs. The populations involved in the included studies in our analysis are relatively heterogeneous and analyze different drugs. Combined outcome analyses may overestimate or underestimate the benefit of the results found. Also, we did not included hard endpoints such as mortality. Further studies are required to establish the efficacy and safety of DOACs in valvular AF and the effects of their use in different valve diseases.

## Conclusion

This study demonstrated that DOACs, compared to Warfarin, in patients with AF and VHD showed a reduction in thromboembolic events (stroke and systemic embolism), with a better safety profile (reduction in intracranial hemorrhage). Individual differences between each drug (rivaroxaban, apixaban, dagigatran, edoxaban) need to be clarified in further studies.

## Data Availability Statement

The original contributions generated for this study are included in the article/Supplementary Material, further inquiries can be directed to the corresponding author/s.

## Author Contributions

YB: conceptualization, methodology, and writing-original draft preparation. AD: supervision, investigation, and writing-original draft preparation. LR and MG: software, visualization, and investigation. LL-K: data curation, writing-review and editing, and validation. EB: writing-reviewing and editing. All authors contributed to the article and approved the submitted version.

## Conflict of Interest

The authors declare that the research was conducted in the absence of any commercial or financial relationships that could be construed as a potential conflict of interest.

## Publisher's Note

All claims expressed in this article are solely those of the authors and do not necessarily represent those of their affiliated organizations, or those of the publisher, the editors and the reviewers. Any product that may be evaluated in this article, or claim that may be made by its manufacturer, is not guaranteed or endorsed by the publisher.
