# Supplementary material for: Comparison of the Direct Oral Anticoagulants and Warfarin in Patients With Atrial Fibrillation and Valvular Heart Disease: Updated Systematic Review and Meta-Analysis of Randomized Controlled Trials
Source: Front Cardiovasc Med. 2021 Sep 22;8:712585. doi: 10.3389/fcvm.2021.712585 (PMC8494252; doi:10.3389/fcvm.2021.712585)
Supplement: Supplementary file 1 [file Data_Sheet_1.docx]

**Comparison of the direct oral anticoagulants and warfarin in patients with atrial fibrillation and valvular heart disease: Updated systematic review and meta‐analysis of randomized controlled trials**

DOACs vs Warfarin in AF with VHD

Yasmin de Souza Lima Bitar, B.S. in health^a,b^, Andre Rodrigues Duraes, MD, PhD ^a,b^, Leonardo Roever, MHS ^c^, Mansueto Gomes Neto, PhD ^a,b^, Liliane Lins-Kusterer, PhD^a,b^, Edimar Alcides Bocchi, MD, PhD ^d,e^

^a^ Post-graduate Program in Medicine and Health, PPgMS/UFBA – Brazil.

^b^ Federal University of Bahia, UFBA – Brazil.

^c^ Federal University of Uberlandia – Brazil

^d^ University of São Paulo Medical School, HCFMUSP - Brazil.

^e^ Heart Institute, InCor - Brazil

Correspondence to Yasmin de Souza Lima Bitar, B.S. in health, medicine student; Address for correspondence: Federal University of Bahia, medical school – UFBA/FAMEB; XV de novembro Square, s/n - Largo do Terreiro de Jesus, 40025-010, Salvador, Bahia, Brazil. E-mail: yasmin.ufba@gmail.com. Telephone number: 55 73 991371560.

**ELECTRONIC SUPPLEMENTARY FILES**

**File 1: Table E1.** PRISMA Checklist

| **Section/topic** | **#** | **Checklist item** | **Reported on page #** |
| --- | --- | --- | --- |
| **TITLE** | | |  |
| Title | 1 | Identify the report as a systematic review, meta-analysis, or both. | 1 |
| **ABSTRACT** | | |  |
| Structured summary | 2 | Provide a structured summary including, as applicable: background; objectives; data sources; study eligibility criteria, participants, and interventions; study appraisal and synthesis methods; results; limitations; conclusions and implications of key findings; systematic review registration number. | 3 |
| **INTRODUCTION** | | |  |
| Rationale | 3 | Describe the rationale for the review in the context of what is already known. | 4 |
| Objectives | 4 | Provide an explicit statement of questions being addressed with reference to participants, interventions, comparisons, outcomes, and study design (PICOS). | 4 |
| **METHODS** | | |  |
| Protocol and registration | 5 | Indicate if a review protocol exists, if and where it can be accessed (e.g., Web address), and, if available, provide registration information including registration number. | - |
| Eligibility criteria | 6 | Specify study characteristics (e.g., PICOS, length of follow-up) and report characteristics (e.g., years considered, language, publication status) used as criteria for eligibility, giving rationale. | 4 |
| Information sources | 7 | Describe all information sources (e.g., databases with dates of coverage, contact with study authors to identify additional studies) in the search and date last searched. | 4 |
| Search | 8 | Present full electronic search strategy for at least one database, including any limits used, such that it could be repeated. | 5 |
| Study selection | 9 | State the process for selecting studies (i.e., screening, eligibility, included in systematic review, and, if applicable, included in the meta-analysis). | 4-5 |
| Data collection process | 10 | Describe method of data extraction from reports (e.g., piloted forms, independently, in duplicate) and any processes for obtaining and confirming data from investigators. | 5 |
| Data items | 11 | List and define all variables for which data were sought (e.g., PICOS, funding sources) and any assumptions and simplifications made. | 5 |
| Risk of bias in individual studies | 12 | Describe methods used for assessing risk of bias of individual studies (including specification of whether this was done at the study or outcome level), and how this information is to be used in any data synthesis. | 5 |
| Summary measures | 13 | State the principal summary measures (e.g., risk ratio, difference in means). | 5 |
| Synthesis of results | 14 | Describe the methods of handling data and combining results of studies, if done, including measures of consistency (e.g., I^2^) for each meta-analysis. | 5 |

| Risk of bias across studies | 15 | Specify any assessment of risk of bias that may affect the cumulative evidence (e.g., publication bias, selective reporting within studies). | 5 |
| --- | --- | --- | --- |
| Additional analyses | 16 | Describe methods of additional analyses (e.g., sensitivity or subgroup analyses, meta-regression), if done, indicating which were pre-specified. | 5 |
| **RESULTS** | | |  |
| Study selection | 17 | Give numbers of studies screened, assessed for eligibility, and included in the review, with reasons for exclusions at each stage, ideally with a flow diagram. | 6 |
| Study characteristics | 18 | For each study, present characteristics for which data were extracted (e.g., study size, PICOS, follow-up period) and provide the citations. | 6 |
| Risk of bias within studies | 19 | Present data on risk of bias of each study and, if available, any outcome level assessment (see item 12). | 7 |
| Results of individual studies | 20 | For all outcomes considered (benefits or harms), present, for each study: (a) simple summary data for each intervention group (b) effect estimates and confidence intervals, ideally with a forest plot. | 6-7 |
| Synthesis of results | 21 | Present results of each meta-analysis done, including confidence intervals and measures of consistency. | 6-7 |
| Risk of bias across studies | 22 | Present results of any assessment of risk of bias across studies (see Item 15). | 6-7 |
| Additional analysis | 23 | Give results of additional analyses, if done (e.g., sensitivity or subgroup analyses, meta-regression [see Item 16]). | 7 |
| **DISCUSSION** | | |  |
| Summary of evidence | 24 | Summarize the main findings including the strength of evidence for each main outcome; consider their relevance to key groups (e.g., healthcare providers, users, and policy makers). | 8 |
| Limitations | 25 | Discuss limitations at study and outcome level (e.g., risk of bias), and at review-level (e.g., incomplete retrieval of identified research, reporting bias). | 10 |
| Conclusions | 26 | Provide a general interpretation of the results in the context of other evidence, and implications for future research. | 11 |
| **FUNDING** | | |  |
| Funding | 27 | Describe sources of funding for the systematic review and other support (e.g., supply of data); role of funders for the systematic review. | 11 |

From: Moher D, Liberati A, Tetzlaff J, Altman DG, The PRISMA Group (2009). Preferred Reporting Items for Systematic Reviews and Meta-Analyses: The PRISMA Statement. PLoS Med 6(6): e1000097. doi:10.1371/journal.pmed1000097. For more information, visit: www.prisma-statement.org.

**File 2.** Search Strategy, Eligibility Criteria, Risk of Bias and Certainty in the evidence and strength of recommendations

**Search Strategy**

We searched PubMed and LILACS, MEDLINE, SciELO, and Cochrane Library (November 2020 – December 2020), updating from 2010 to 2020. For additional data collection, we also reviewed sites of the pharmaceutical industries and the lists of references of the selected publications. A data extraction form was prepared for the retrieval of information, such as: year of publication, authors, type of RCTs, main characteristics of the population involved, types of VHD included and excluded in the study, type of administered DOAC and doses, outcomes (total efficacy rate and safety), and follow-up time. The data was extracted and summarized by the authors, independently.

Electronic searches were carried out with Boolean operators (AND and OR) and keywords defined by MeSH (Medical Subject Headings Terms) and other words suggested by the authors. The following search terms were used: "NOACS" [Author's terms], "DOACS" [Author's terms], "NVKA" [Author's terms], "Apixaban" [Mesh terms], "Rivaroxaban" [Mesh terms], "Dabigatran" [Mesh terms], "Edoxaban" [Mesh terms], "Betrixaban" [Mesh terms], "Warfarin" [Mesh terms], “VKA” [Author's terms], “anticoagulants” [Mesh terms], “drug therapy” [Mesh terms], “treatment outcome” [Mesh terms], “valvular heart disease” [Mesh terms], “VHD” [Author's terms], “heart valve disease” [Mesh terms], “valvular atrial fibrillation” [Author's terms], “atrial fibrillation" [Mesh terms], "bioprothesis" [Author's terms], "heart valve prostheses" [Mesh terms], "mechanical heart valves" [Author's terms] and "stroke” [Mesh terms].

For the refinement of the search, we combined the descriptors. Example search in PUBMED: 1) (((apixaban) AND (warfarin)) AND (mechanical heart valves)) OR (bioprostheses); (((dabigatran) AND (warfarin)) AND (mechanical heart valves)) OR (bioprostheses); (((edoxaban) AND (warfarin)) AND (mechanical heart valves)) OR (bioprostheses); (((rivaroxaban) AND (warfarin)) AND (mechanical heart valves)) OR (bioprostheses), and others. Language was not restricted.

All the search results were downloaded for further evaluation. Two of the authors (Bitar, Y. and Duraes, A) performed the screening of titles and abstracts - a table with the English translation of all the titles and the English abstracts was reviewed. Both authors reviewed full-text articles, and determined their eligibility to access the trials for inclusion. The divergences were resolved by consensus discussion with a third author (Neto, M).

**Eligibility Criteria**

The inclusion criteria were randomized clinical trials that analyzed the effects on efficacy - in the reduction of stroke and ES events - and safety - in reducing major bleeding rates and intracranial hemorrhage -, regarding the use of DOACs (Dabigatran, Rivaroxaban, Apixaban, Edoxaban, Betrixaban), in comparison to the Warfarin in adult humans with AF and VHD (including MHV - with ≥ three months postoperatively - and bioprostheses).

**Risk of Bias in Individual Studies**

The modified Cochrane risk‐of‐bias tool evaluated the risk of bias and methodological quality assessment of included trials. The following domains were evaluated: **selection bias (**random sequence generation method and allocation concealment); **performance bias** (blinding of participants and personnel); **detection bias** (blinding of outcome assessment); **attrition bias** (incomplete outcome data); and **reporting bias** (selective reporting).(1) The quality of each item was classified using a nominal scale: “Yes” (low risk of bias), “No” (high risk of bias) or “Unclear” (unclear risk of bias).

**Certainty in the evidence and strength of recommendations**

In our meta-analysis, we assessed the certainty of evidence and strength of recommendations for the outcomes of stroke and SE composition, the presence of major bleeding and intracranial hemorrhage after the use of DOACs and Warfarin by using the GRADEpro (Grading of Recommendations, Assessment, Development and Evaluation profiler) software.(2) The GRADE approach classifies evidence as high, moderate, low, or very low quality based on the following considerations: risk of bias, consistency, directness, precision, and publication bias.(3) The quality of evidence was interpreted as high quality, moderate quality, low quality or very low quality.

**File 3. Table E1.** Summary of the main characteristics of studies involving the use of DOACs in patients with AF and VHD included in the present systematic review. (n = 8)

| Reference | Study Design | Intervention | Sample / Randomization | VHD |
| --- | --- | --- | --- | --- |
| Eikelboom et al.2013(4)  (RE-ALIGN) | RCT Phase II clinical trial, dose-validation, prospective, open-label.  **Follow-up: 12 weeks** | Dabigatran 150, 220 or 300 mg BID (according to CrCl), with adjusted doses to obtain plasma levels higher than at least 50ng per milliliter; or Warfarin adjusted by INR. | 252 patients; 168 used Dabigatran and 84 Warfarin. Population A = 199 patients, in early postoperatively (133 in DG and 66 in VG), and population B = 53 patients in the late postoperatively (35 in DG and 18 GV). | 252 MHV; in aortic position in 172 patients (68%), mitral in 71 (28%), in both 9 (4%). |
| Breithardt et al.2014(5)  (ROCKET-AF) | Post-hoc analysis of a RCT phase III, prospective, double-blind, double-masked, multicenter. **Follow-up: 1.9 years** | Rivaroxaban 20 mg QD (or 15 mg QD with CrCl 30-49 mL/min) or Warfarin according to INR (adjusted for 2-3). | 1992 recruited. 939 used Rivaroxaban (49% AS, 48% MR or AR); 1001 used Warfarin (51% AS, 52% MR or AR); 52 not informed. † | MR 1756 (89.6%), AR 486 (24.8%), AS 215 (11%), other 11 (0.6%). † |
| Avezum et al.2015(6)  (ARISTOTLE) | Post-hoc analysis of a phase III RCT prospective, double-blind, double-masked, multicenter.  **Follow-up: 1.8 years** | Apixaban 5 mg BID (2.5 mg if ≥ 2 of the following: age ≥80 years, weight ≤60 kg, Cr ≥1.5 mg/dL) or Warfarin (according to INR setting for 2-3). | 4808 recruited. ‡ 2438 used Apixaban (72.9% had MR, 2.8% MS, 19% AR and 8.5% AS, 44.4% TR); 2370 used Warfarin. | MR 3,526 (73.3%), TR 2124 (44.2%), AR 887 (18.4%), AS 384 (8%), MS 131 (2.7%), previous valve surgery 251 (5.2%). |
| Duraes et al.2016(7)  (DAWA) | This is a prospective phase II RCT, pilot, open-label  **Follow-up: 12 weeks** | Dabigatran 110mg BID or Warfarin according to INR. | 27 patients recruited; 15 received Dabigatran and 12 Warfarin. | Mitral or aortic bioprosthesis; 11 in mitral position in the Dabigatran group, and 9 from the Warfarin group |
| Ezekowitz et al.2016(8)  (RE-LY) | Post-hoc analysis of a RCT III, open, prospective, partially blind, multicenter.  **Follow-up: 2 years** | Dabigatran 110 mg BID or Dabigatran 150 mg BID, or Warfarin (as adjusted from INR  to 2-3). | 3,950 patients recruited; Uninformed randomization in patients with VHD. * | MR 3101 (17.1%), TR 1179 (6.5%), AR 817 (4.5%), AS 471 (2.6%), mild MS 193 (1.1%) |
| De Caterina et al.2017(9)  (ENGAGE AF-TIME 48) | Post-hoc analyzes of a phase III RCT, double-blind.  **Follow-up: 2.8 years** | Edoxaban 60 mg QD (30 mg QD if ≥ 1 of the criteria: CrCl 30-50 ml/min, ≤60 kg, or concomitant therapy with P-gp inhibitors) or Edoxaban 30 mg QD (or 15 mg QD with ≥ 1 of the previous criterion) or Warfarin according to INR. | 2.824 patients recruited; Uninformed randomization in patients with VHD. | 191 patients with bioprosthesis; aortic position (31.4%), mitral position (68.6%). MR, 2.250 (10.7%); 369, AR (17%); AS, 165 (0.8%); valvular repair surgery, 123 (0.6%); valvuloplasty, 19 (0.9%). |
| Durães et al.2020^22^  (RIWA) | This is a prospective phase II RCT, pilot, open-label **Follow-up: 12 weeks** | Rivaroxaban 15mg BID or Warfarin according to INR. | 44 patients recruited; 23 received Rivaroxaban and 21 Warfarin. | Mitral or aortic MHV; 26 isolated mitral; 8 isolated aortic; 10 mitroaortic. |
| Guimarães et al. 2020^23^  (RIVER) | This is a prospective phase III RCT, open-label, multicenter, with blinded adjudication of outcomes **Follow-up: 1 year** | Rivaroxaban 20mg QD (CrCl of 30-49 ml/min received a reduced dose of 15 mg QD) or Warfarin according to INR. | 1005 patients recruited; 500 received Rivaroxaban and 505 Warfarin | Bioprosthetic mitral valve and permanent, paroxysmal, or persistent AF or flutter. |

AF = atrial fibrillation; VHD = valvular heart disease; NOAC = new oral anticoagulant; MHV = mechanical heart valves; CrCl= creatinine clearence; INR = international normalized ratio; TE = thromboembolic; VKA =vitamin K antagonist; RCT = randomized clinical trial; AF = atrial fibrillation; BID = twice daily; QD = once a day; SD = standard deviation; AS = aortic stenosis; MS = mitral stenosis; MR = mitral regurgitation; AR = aortic regurgitation; TR = tricuspid regurgitation. * The authors did not discriminate the randomization performed in patients with VHD because they were not objectives of the original studies; however, the authors report that 485 female subjects with VHD used Dabigatran 110mg, and 560 of the same gender used Dabigatran 150mg, while 562 remaining were randomized to the Warfarin group. † In the original study, it is known that 2003 involved patients had VHD, however, eleven of these patients were in a place where they violated good clinical practice guidelines and four additional patients were randomized but did not receive study drugs. Because of this, only 1992 patients were used in the analysis of the outcomes to the use of Rivaroxaban, in a study by Breithardt et al. (2014). However, a later study conducted by these authors (2016), it was evidenced that 52 patients with VHD, the valve site was unknown. Because of this, the results of clinical outcomes and efficacy analyzes were omitted, leaving only 1940 patients.

**File 4. Table E2.** Summary of the main clinical characteristics and risk Factors for bleeding and thromboembolic events in patients with AF and VHD involved in the studies included in the present systematic review. (n = 8)

| Authors /  Year | *N* | *Age*  *(mean)* | *Gender*  *N (%)* | *CHA2DS2-VASc (mean)* | *HAS-BLED (mean)* | *Comorbidities and Risk Factors* |
| --- | --- | --- | --- | --- | --- | --- |
| Eikelboom et al.2013(4)  (RE-ALIGN) | 252 | 55.8 ±9.9 | 89 (35.3) F;  163 (64.7) M. | Not identified | Not identified | SAH (61.1); Dyslipidemia (46.4); NYHA ≥ II (36.1); CAD (25); DM (15.8). |
| Breithardt et al.2014(5)  (ROCKET-AF) | 2003 | 75 | 785 (39.4) F;  1207 (60.6) M. | 3.5 | 2.8 | SAH (89.1); HF (70.4); CrCl 62 mL/min (49.8); prior stroke embolism or TIA (48.2); DM (40.1). |
| Avezum et al.2015(6)  (ARISTOTLE) | 4.808 | 71 | 1936 (40.3) F;  2872 (59.7) M. | 2.2 | Not identified | SAH (85.3); HF (48.6); DM (22.6); prior stroke, embolism or TIA (18.8); mild renal insufficiency (43.8). |
| Duraes et al.2016(7)  (DAWA) | 27 | 47.2±8.2 | 17 (62.9) F;  10 (37.1) M. | Not identified | 0 (in both groups). | DG vs WG: SAH (46.7 vs 50); prior stroke (26.7 vs 33.3); smoking (13.3 vs 35); DM (7.1 vs none). |
| Ezekowitz et al.2016(8)  (RE-LY) | 3.950 | 74 | 1607 (40.7%) F;  2334 (59.3%) M. | 2 | Not identified | SAH (77.2); history of HF (39.7); CAD (32.5); prior stroke/SE/TIA (22.2); moderate renal insufficiency (21.8); prior AMI (18.1). |
| De Caterina et al.2017(9)  (ENGAGE  AF-TIME 48) | 2.824 | 71.8 ±9.4 | 1.193 (42.2%) F;  1.631 (57.8%) M. | 4.56±1.43 | 2.55±0.98 | SAH (93.1); HF (73.7); CAD (39.8); DM (32.2); prior stroke/TIA (23.7). |
| Durães et al.2020^22^  (RIWA) | 44 | 44.2 ±9.5 | 27 F (61.3) F;  17 (38.7) M. | 2.3±1.1 | 1.8±0.97 | Previous rheumatic fever (26); AF (12); SAH (32); Previous stroke/TIA (10). |
| Guimarães et al. 2020^23^  (RIVER) | 1005 | 59.3 ±12.2 | 607 (60.4) F;  398 (39.6) M. | 2.6±1.4 | 1.6±0.9 | SAH (60.7); HF (38.8); Dyslipidemia (33.6); DM (13.7); Previous stroke (12.8) |

N = number; VHD = valvular heart disease; NOAC = new oral anticoagulant; CrCl =creatinine clearence; TE = thromboembolism; AF = atrial fibrillation; DG = Dabigatran group; WG = Warfarin group; M = male; F = female; SAH = systemic arterial hypertension; HF = heart Failure; DM = diabetes mellitus; SE = systemic embolism; TIA = transient ischemic event; NYHA = functional classification of the New York Heart Association; CAD = coronary artery disease; AMI = acute myocardial infarction**.**

**File 5: Figure E1.** Risk of bias summary for each included study according to the Cochrane Collaboration Tool’s classification.


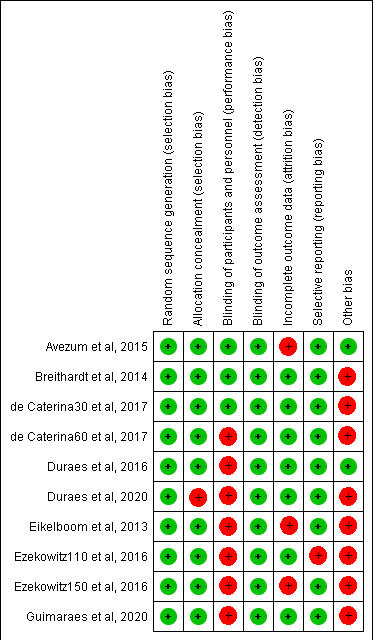


The red circle indicates uncertain data and the green circle low risk of bias.

**File 6: Table E3.** GRADE assessments (summary of findings) – Valvular Heart Disease.

|  | | | | | | |
| --- | --- | --- | --- | --- | --- | --- |
| **DOACs compared to Warfarin for Valvular Heart Disease** | | | | | | |
| **Patient or population**: Valvular Heart Disease  **Setting**:  **Intervention**: DOACs  **Comparison**: Warfarin | | | | | | |
| Outcomes | **Anticipated absolute effects^*^** (95% CI) | | Relative effect (95% CI) | № of participants  (studies) | Certainty of the evidence (GRADE) | Comments |
|  | **Risk with Warfarin** | **Risk with DOACS** |  |  |  |  |
| Stroke or systemic embolism | 35 per 1.000 | **28 per 1.000** (24 to 33) | RR 0.80 (0.68 to 0.94) | 18686 (8 RCTs) | ⨁⨁⨁◯ MODERATE ^a^ |  |
| Intracranial Haemorrhage | 15 per 1.000 | **6 per 1.000** (4 to 10) | RR 0.40 (0.24 to 0.66) | 15487 (6 RCTs) | ⨁⨁◯◯ LOW ^a,b^ |  |
| Major Bleeding | 61 per 1.000 | **51 per 1.000** (34 to 76) | RR 0.83 (0.56 to 1.24) | 16826 (7 RCTs) | ⨁⨁◯◯ LOW ^a,b^ |  |
| ***The risk in the intervention group** (and its 95% confidence interval) is based on the assumed risk in the comparison group and the **relative effect** of the intervention (and its 95% CI).   **CI:** Confidence interval; **RR:** Risk ratio | | | | | | |
| **GRADE Working Group grades of evidence** **High certainty:** We are very confident that the true effect lies close to that of the estimate of the effect **Moderate certainty:** We are moderately confident in the effect estimate: The true effect is likely to be close to the estimate of the effect, but there is a possibility that it is substantially different **Low certainty:** Our confidence in the effect estimate is limited: The true effect may be substantially different from the estimate of the effect **Very low certainty:** We have very little confidence in the effect estimate: The true effect is likely to be substantially different from the estimate of effect | | | | | | |

#### Explanations

a. Studies without allocation concealment, blinding, and/or sample size calculation.

b. Meta-analysis with statistical significance in heterogeneity test and high I2.

**File 7: Table E4.** GRADE assessments (summary of findings) - Bioprosthetic heart valves.

|  | | | | | | |
| --- | --- | --- | --- | --- | --- | --- |
| **DOACs compared to Warfarin for Valvular Heart Disease** | | | | | | |
| **Patient or population**: Bioprosthetic heart valves  **Setting**:  **Intervention**: DOACs  **Comparison**: Warfarin | | | | | | |
| Outcomes | **Anticipated absolute effects^*^** (95% CI) | | Relative effect (95% CI) | № of participants  (studies) | Certainty of the evidence (GRADE) | Comments |
|  | **Risk with Warfarin** | **Risk with DOACS** |  |  |  |  |
| Stroke or systemic embolism | 43 per 1.000 | **20 per 1.000** (11 to 38) | RR 0.47 (0.26 to 0.88) | 1449 (5 RCTs) | ⨁⨁⨁◯ MODERATE ^a^ |  |
| Intracranial Haemorrhage | 12 per 1.000 | **2 per 1.000** (0 to 13) | OR 0.18 (0.03 to 1.05) | 1161 (2 RCTs) | ⨁⨁⨁◯ MODERATE ^a^ |  |
| Major Bleeding_ | 55 per 1.000 | **27 per 1.000** (16 to 46) | RR 0.49 (0.29 to 0.83) | 1447 (5 RCTs) | ⨁⨁⨁◯ MODERATE ^a^ |  |
| ***The risk in the intervention group** (and its 95% confidence interval) is based on the assumed risk in the comparison group and the **relative effect** of the intervention (and its 95% CI).   **CI:** Confidence interval; **RR:** Risk ratio; **OR:** Odds ratio | | | | | | |
| **GRADE Working Group grades of evidence** **High certainty:** We are very confident that the true effect lies close to that of the estimate of the effect **Moderate certainty:** We are moderately confident in the effect estimate: The true effect is likely to be close to the estimate of the effect, but there is a possibility that it is substantially different **Low certainty:** Our confidence in the effect estimate is limited: The true effect may be substantially different from the estimate of the effect **Very low certainty:** We have very little confidence in the effect estimate: The true effect is likely to be substantially different from the estimate of effect | | | | | | |

#### Explanations

a. Studies without allocation concealment, blinding, and/or sample size calculation.

**File 8. Table E5**. Summary of the main pharmacological characteristics of DOACS approved by FDA for use in U.S.

| CHARACTERISTICS | DABIGATRAN | RIVAROXABAN | APIXABAN | EDOXABAN | BETRIXABAN |
| --- | --- | --- | --- | --- | --- |
| **Commercial name** | Pradaxa® | Xarelto® | Eliquis® | Savaysa® | Bevyxxa® |
| **Dosage forms (mg)** | Capsule 75 and 150 * | Tablet 2.5, 10, 15 and 20 * | 2.5 and 5 * | Tablet 15, 30 and 60 * | Capsule 40 and 80 |
| **Mechanism of action** | Direct Thrombin Inhibitor | Factor Xa Inhibitors | Factor Xa Inhibitors | Factor Xa Inhibitors | Factor Xa Inhibitors |
| **Metabolism** | By P-gp and CYP3A4 | By P-gp and CYP3A4 | By P-gp and CYP3A4 | By P-glycoprotein | CYP-independent hydrolysis |
| **Excretion** | 80% urine; 20% hepatic. | 1/3 urine and 2/3 feces. | 3/4 feces and 1/4 urine. | 50% urine | 85% feces and 11% urine |
| **Bioavailability (%)** | ~ 3 - 7 | ~ 80 - 100 | ~ 50 | 62 | 34 |
| **PPT (hr)** | 1 | 2- 4 | 3 - 4 | 1 - 2 h | 3 - 4 |
| **Half-life (hr)** | 12 - 17 § | 5 – 9 or 11 - 13 (elderly) | 5 – 6 (dominant) | 10 - 14 | 19 - 27 |
| **Indications** | Stroke/SE prophylaxis with AF; DVT or PE treatment or profilaxis | DVT or PE treatment or prophylaxis; Nonvalvular AF; Reduction of RMCE | Stroke prophylaxis with AF; DVT or PE treatment or prophylaxis; Nonvalvular AF | Stroke prophylaxis with AF; DVT or PE treatment; Nonvalvular AF | DVT Prevention |
| **Renal impairment** | CrCl ≥ 15 to < 30: necessary adjustment and < 15 mg/dL: avoid use | CrCl ≥ 15 to < 30: closely monitor and < 15 mg/dL or ESRD on dialysis: avoid use | Necessary adjustment if Cr ≥1.5mg/dL; CrCl < 15 mg/dL or ESRD on dialysis: avoid | CrCl 15 to < 50: necessary adjustment and >90 mg/dL: do not use (in NVAF) | CrCl ≥ 15 to < 30 mL/min: initial dose 80mg, then 40mg QD |
| **Hepatic impairment** | Moderate to severe: avoid | Moderate to severe: avoid | Moderate to severe: avoid | Moderate to severe: avoid | Moderate to severe: avoid |
| **Dose modifications** | Renal impairment, P-gp inhibitor coadministration | Renal impairment | Renal impairment, age, body weight | Renal impairment, weight | Renal impairment, P-gp inhibitor coadministration |
| **Reverser** | Idarucizumabe (Praxbind®) | Coagulation FXa - Recombinat (Andexxa®) | Coagulation FXa - Recombinat (Andexxa®) | Not available. | Not available. |
| Drug interactions | P-gp inhibitors or inducers | P-gp and CYP3A4 inhibitors or inducers | P-gp and CYP3A4 inhibitors or inducers | P-gp inhibitors | P-gp inhibitors |

AF = atrial fibrillation; DOAC = direct oral anticoagulant; FDA = Food and Drug Administration; CrCl = creatinine clearence; PPT = peak plasma time; PE = pulmonary embolus; P-gp = P-glycoprotein; FXa = factor Xa Inhibitors; NVAF = Nonvalvular atrial fibrillation; DVT = deep vein thrombosis; RMCE = risk of major cardiovascular events; NR = not recommended; BID = twice daily; QD = once a day; HR = hour; * The indication of the use of a given presentation varies according to the clinical indication and renal adjustment when necessary; ** Major adverse events often reported in addition to bleeding;§If normal renal function.

**REFERENCES**

1. Higgins J, Altman D, Gotzsche P, Juni P, Moher D, Oxman A, et al. Chapter 8: Assessing Risk of Bias in Included Studies. Cochrane Handbook for Systematic Reviews of Interventions Version 5.1.0. Cochrane Collab. 2011;

2. Schünemann HJ, Schünemann AHJ, Oxman AD, Brozek J, Glasziou P, Jaeschke R, et al. Grading quality of evidence and strength of recommendations for diagnostic tests and strategies. BMJ. 2008 May 17;336(7653):1106–10.

3. Guyatt GH, Oxman AD, Kunz R, Falck-Ytter Y, Vist GE, Liberati A, et al. Going from evidence to recommendations. BMJ. 2008 May 10;336(7652):1049–51.

4. Eikelboom JW, Connolly SJ, Brueckmann M, Granger CB, Kappetein AP, Mack MJ, et al. Dabigatran versus warfarin in patients with mechanical heart valves. N Engl J Med. 2013 Sep 26;369(13):1206–14.

5. Breithardt G, Baumgartner H, Berkowitz SD, Hellkamp AS, Piccini JP, Stevens SR, et al. Clinical characteristics and outcomes with rivaroxaban vs. warfarin in patients with non-valvular atrial fibrillation but underlying native mitral and aortic valve disease participating in the ROCKET AF trial. Eur Heart J. 2014 Dec 14;35(47):3377–85.

6. Avezum A, Lopes RD, Schulte PJ, Lanas F, Gersh BJ, Hanna M, et al. Apixaban Compared with Warfarin in Patients With Atrial Fibrillation and Valvular Heart Disease: Findings From the ARISTOTLE Trial. Circulation. 2015;

7. Rodrigues Durães A, Pollianna de Souza Roriz B, Bianca de Almeida Nunes B, Pinho Albuquerque F, Fábio Vieira de Bulhões B, Andre Mauricio de Souza Fernandes B, et al. Dabigatran Versus Warfarin After Bioprosthesis Valve Replacement for the Management of Atrial Fibrillation Postoperatively: DAWA Pilot Study. Drugs R D. 2016;16.

8. Ezekowitz MD, Nagarakanti R, Noack H, Brueckmann M, Litherland C, Jacobs M, et al. Comparison of Dabigatran and Warfarin in Patients With Atrial Fibrillation and Valvular Heart DiseaseClinical Perspective. Circulation. 2016 Aug 23;134(8):589–98.

9. De Caterina R, Renda G, Carnicelli AP, Nordio F, Trevisan M, Mercuri MF, et al. Valvular Heart Disease Patients on Edoxaban or Warfarin in the ENGAGE AF-TIMI 48 Trial. J Am Coll Cardiol. 2017 Mar 21;69(11):1372–82.
